# Magnetic States in Ensemble of Ferromagnetic Nanoparticles in Cu-Mn-Al Alloy

**DOI:** 10.1186/s11671-016-1233-z

**Published:** 2016-01-14

**Authors:** S. M. Konoplyuk, L. E. Kozlova, V. V. Kokorin, A. O. Perekos, O. V. Kolomiets

**Affiliations:** Institute of Magnetism, National Academy of Science of Ukraine and Ministry for Education and Science of Ukraine, Vernadsky Blvd. 36-b, Kyiv, 03680 Ukraine; G.V. Kurdyumov Institute for Metal Physics, National Academy of Science of Ukraine, Vernadsky Blvd. 36, Kyiv, 03680 Ukraine; Department of Physics, Lviv Polytechnic National University, S. Bandery Str. 12, Lviv, 79013 Ukraine

**Keywords:** Granular system, Superparamagnetism, Magnetoresistance, Cu-Mn-Al

## Abstract

Two Cu-Mn-Al samples of different compositions were studied: one exhibiting martensitic transformation, another without structural transition. X-ray diffraction and magnetic measurements demonstrate that different magnetic behaviors of alloys originate from different concentrations and sizes of ferromagnetic nanoparticles, which appear after solid solution decomposition.

Estimation of magnetic moments of ferromagnetic nanoparticles from magnetization curves was performed using Langevin function and compared to those obtained from X-ray examination. Granular systems are known to show giant magnetoresistance. Therefore, magnetoresistance of Cu-Mn-Al melt-spun ribbons after different aging times was measured. The study has shown that increase in the concentration of Mn atoms and time of aging in Cu-Mn-Al alloy leads to an increase in the amount of precipitated phase appearing as ferromagnetic nanoparticles.

## Background

Magnetic phenomena related to spin-dependent electron scattering attract great attention as their study forms scientific basis for emerging technologies of ultrahigh-density recording and fabrication of magnetic sensors. Generally speaking, there are two main types of materials displaying spin-dependent scattering—multilayers and nanogranular systems [1]. From the practical point of view, the most prospective is utilization of multilayers as they contain specific combination of layers tailored for concrete practical applications. Nanoscale granular systems including ensemble of ferromagnetic nanoparticles dispersed in nonmagnetic or antiferromagnetic matrix are of less practical interest, but they are interesting for fundamental science. The magnetic moments of these particles often referred to as “superspins” are dissipation centers for moving electrons. The magnetic field affects direction of “superspins” decreasing scattering of electrons and hence reducing electric resistance. This phenomenon called giant magnetoresistance, as well as other magnetic and transport properties of the nanogranular system, depends on a variety of parameters such as size, density, and magnetic moment of nanosized particles. They can couple each other by dipolar, exchange, or RKKY interactions [2]. Collective behavior of nanoparticles can be modified to some extent by thermal treatment.

One of the alloys where formation of microstructure mentioned above occurs is Cu-Mn-Al one. In the Cu-Mn-Al system, there is a miscibility gap—the range of compositions wherein solid solution decomposition occurs. In this range according to [3], decomposition leads to appearance of ferromagnetic Cu_2_MnAl and nonmagnetic Cu_3_Al phases from solid solution below 623 K: the former has L2_1_-ordered structure, the latter has DO_3_-ordered one. It was reported [4] that GMR in the Cu-Mn-Al system can achieve 15 %.

In other works, martensitic transformation occurring in this alloy was investigated. Results demonstrated that characteristic temperatures of the transformation decreased as Mn content was increased. In this system, Mn atoms play a significant role as only they possess magnetic moments providing ferromagnetism of Cu_2_MnAl compound. Therefore, changing concentration of Mn atoms, one can govern magnetic and transport properties of decomposed Cu-Mn-Al alloy. In this study, influence of Mn concentration and thermal treatment on the structure and properties of Cu-Mn-Al alloys was investigated.

## Methods

The Cu-Mn-Al ribbons of two compositions were melt-spun: Sp1 (Cu-5 % Mn-23.4 % Al) and Sp2 (Cu-7 % Mn-26 % Al). Before crushing for melt spinning, the ingots were prepared by induction melting in argon atmosphere. Melt spinning was carried out by dropping the melt onto a copper wheel rotating with the velocity of 25 m/s. The fabricated ribbons were 100 μm thick. They were aged for 1, 2, 6, and 12 h to obtain different parameters of dispersed particles. Magnetization of ribbons was measured by the extraction technique using the commercial Physical Property Measurement System (PPMS) from Quantum Design. The samples for the magnetization measurements were prepared by crushing the Cu-Mn-Al ribbons into submillimeter parts and compacting them randomly in a gelatin capsule. Magnetoresistance was measured by four-terminal method along the direction of magnetic field and calculated as ratio *ρ* = (*R*(*H*) − *R*(0))/*R*(0) × 100 %, where *H* is the magnetic field and *R*(0) is the electric resistance of ribbon in zero field. X-ray diffraction patterns were obtained using CuKα radiation for Sp1 and CoKα for Sp2.

## Results and Discussion

Melt spinning is the process in which melted alloy undergoes very fast cooling similar to quenching. It favors formation of a metastable state involving matrix and precipitated nanoparticles. For low content of Mn, decomposition along the Cu_3_Al-Cu_2_MnAl composition line yields formation of Cu_2_MnAl nanoparticles in Cu_3_Al matrix. X-ray diffraction is unable to determine the presence of precipitated particles if they are too small (less than several nanometers). But, aging promotes precipitation and growth of nanoparticles, and after this procedure, X-ray diffraction can detect them in structure. The Sp1 ribbon examined in this experiment was aged at 473 K for 2 h while the Sp2 one experienced the same treatment for 12 h.

**Fig. 1:**
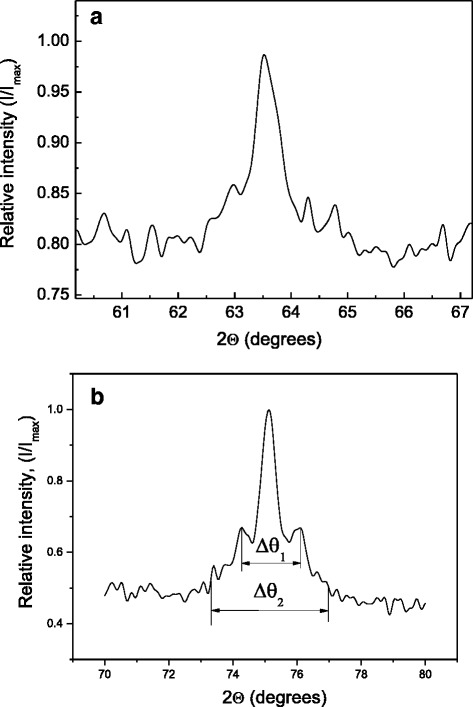
XRD scan: **a** Cu-5 % Mn-23.4 % Al (CuKα radiation) and **b** Cu-7 % Mn-26 % Al (CoKα radiation) alloys near the (400) reflection. Δ*θ*_1_ angular inter-satellite spacing, Δ*θ*_2_ angular width of (400) reflection with satellites

It is known [5] that precipitated coherent Cu_2_MnAl nanoparticles form peculiar quasi-lattice with minimal distance along <100> directions. In Fig. 1, one can see diffraction patterns of the Sp1 (Fig. 1a) and the Sp2 (Fig. 1b) samples near the (400) reflection belonging to the DO_3_ phase. The intensity of diffuse scattering, arising due to static displacements of atoms, increases when approaching to the main reflection. But in contrast to Fig. 1a, Fig 1b shows that the intensity growth is changed into its drop in the vicinity of the (400) maximum. This creates specific signature appearing as two satellite peaks around the main one. It is characteristic of microstructure to comprise matrix and inclusions, situated in correlated positions and creating static displacements in the matrix. In decomposed solid solutions, inclusions imply precipitated particles; thus, the granular system is confirmed to appear in the Sp2 alloy. Diffraction pattern of the Sp1 does not include any remarkable signs of diffuse maxima near the (400) reflection that is probably caused by a very small size of the precipitated particles. Using diffraction pattern of the Sp2, one can estimate average particle diameter *d* and distance *L* between centers of particles in the approximation that they have spherical shape:

1 $$ L = \lambda /2\Delta {\theta}_1 \cos \theta $$

2 $$ d = \lambda /2\Delta {\theta}_2 \cos \theta $$

where *λ* is the radiation wavelength, *θ* is the Bragg angle of the reflection, Δ*θ*_1_ is the angle between centers of satellites, and Δ*θ*_2_ is the angular width of the main peak with satellites.

Substituting values taken from Fig. 1b into (1) and (2), one can obtain 5 ± 1 nm as an average diameter of particle and 7 ± 1 nm as an average distance between the centers of particles. These values allow one to determine the concentration *N* and the volume fraction *p* of precipitated particles: *N* = 2.9 × 10^18^, *p* ~ 0.2. The estimated fraction *p* ~ 0.2 is consistent with that from the Cu_3_Al-Cu_2_MnAl phase diagram for particular alloy composition [3].

There are two basic states in the system of ferromagnetic nanoparticles in nonmagnetic matrix—superparamagnetic and superferromagnetic ones. Occurrence of the superferromagnetic state is attributed to interaction between nanoparticles leading to correlation between orientations of their magnetic moments. As it follows from estimations, the average distance between surfaces of inclusions (roughly 2 nm) excludes direct exchange interaction. In order to find out if other possible interactions are present in the ensemble of nanoparticles or its behavior is clearly superparamagnetic, magnetic measurements were carried out.

**Fig. 2:**
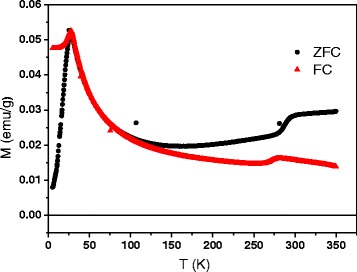
The temperature dependence of ZFC and FC magnetizations for the as-spun Cu-5 % Mn-23.4 % Al alloy in the field of 50 Oe

**Fig. 3:**
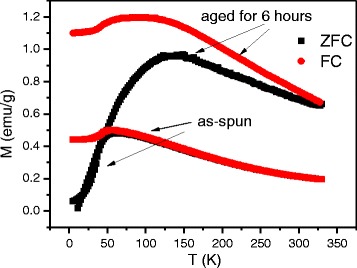
The temperature dependence of ZFC and FC magnetizations for the as-spun and aged Cu-7 % Mn-26 % Al alloys in the field of 50 Oe

The principal criterium to confirm superparamagnetic behavior of nanoparticles is magnetization curve without any remnant magnetization or coercive force. Additional criteria are related to temperature behavior of magnetization. In Figs. 2 and 3, the temperature dependencies of zero-field-cooled (ZFC) and field-cooled (FC) magnetizations in the field of 50 Oe are shown. The as-spun Sp1 ribbon (Fig. 2 ) magnetization is characterized by increase and drop of magnetization on heating and cooling, respectively, at a temperature about 250 K. This feature indicates martensitic transformation in the Cu_3_Al matrix of the alloy that creates elastic stresses in ferromagnetic particles and, in this way, contributes magnetization. In the Sp2 (Fig. 3), higher content of manganese changes ratio of copper to aluminum in the Cu_3_Al matrix bringing the alloy composition out of the range, wherein martensitic transformation is possible. All ZFC curves in Figs. 2 and 3 demonstrate cusp at low temperatures: very sharp for the Sp1 and smoother for the Sp2 ribbons. At lower temperatures, magnetization drops to almost zero. The temperature, where FC and ZFC branches of magnetization curves split, is called blocking temperature *T*_b_. It indicates freezing of magnetic moments of nanoparticles below this temperature. One can see that *T*_b_ of the as-spun Sp1 ribbon is about 30 K while that of the as-spun Sp2 ribbon is about 50 K. It is difficult to determine *T*_b_ for the aged Sp2, since both the ZFC and FC curves slowly approach each other on heating, merging finally above room temperature.

Blocking can originate from two reasons: individual blocking of magnetic moment of nanoparticle in the anisotropy field and the appearance of interaction between particles. Using expression *T*_b_ = *kV*/25*k*_B_, where *k* is the magnetic anisotropy constant, *V* is the volume of particle, and *k*_B_ is the Boltzmann constant, one can find that the blocking temperature due to anisotropy for Cu_2_MnAl particle of 5-nm diameter is less than 1 K. Hence, the first possible reason of “superspin” freezing can be excluded from consideration and, for all ribbons studied, the blocking temperature marks the onset of interparticle interaction on cooling.

**Fig. 4:**
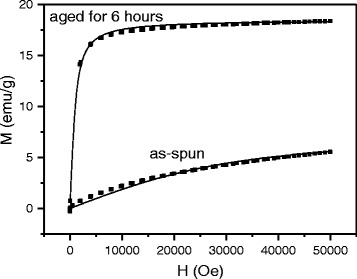
The magnetization as a function of magnetic field for the as-spun and aged Cu-7 % Mn-26 % Al alloys. *Solid lines* are Langevin fittings to magnetization data

In order to establish magnetic state of the ribbons at room temperature and determine parameters of nanoparticle ensemble, additional magnetization measurements were executed. As it follows from Fig. 4, the coercive force of the Sp2 ribbons is equal to zero that implies the superparamagnetic state realizes at room temperature. Taking into account slow approaching both branches on heating (Fig. 3), this behavior can be attributed to weakening dipole-dipole interaction, which is proportional to magnetic moments of particles also decreasing with temperature.

It is known that ferromagnetic nanoparticles measured less than 20 nm in diameter can be described as monodomain particles. The system of such particles in the superparamagnetic regime can be treated using Langevin expression:

3 $$ M/{M}_{\mathrm{S}} = \coth \left(\mu H/{k}_{\mathrm{B}}T\right)\ \hbox{--}\ {k}_{\mathrm{B}}T/\mu H $$

where *M*_S_ is the saturation magnetization, *μ* is the magnetic moment of particle, and *k*_B_ is the Boltzmann constant. Substitution of the experimental data from Fig. 4 into formula (3) and fitting to Langevin function give *μ* as 350 Bohr magneton (*M*_B_) for the as-spun Sp2 and 6000 *M*_B_ for the aged Sp2 ribbon.

According to [6], the atomic magnetic moment of Cu_2_MnAl is about 3 *μ*_B_ per Mn atom and the unit cell measuring 0.59 × 0.59 × 0.59 nm^3^ incorporates four of them. This yields an average diameter of Cu_2_MnAl nanoparticles as 2.2 ± 0.5 and 5.5 ± 0.5 nm for the as-spun and aged Sp2 ribbons, respectively. Using saturation magnetization and calculated above parameters of the granular systems, one can easily estimate the concentration of nanoparticles and the interparticle distance: *N* = 1.35 × 10^19^, *L* = 4 nm for the as-spun alloy and *N* = 2.4 × 10^18^, *L* = 7.5 nm for the alloy aged for 6 h. The concentration and size of particles allowed to determine their volume fractions: 0.9 and 0.21, respectively. Lower value for the as-spun Sp2 ribbon implies that formation of phase precipitates after melt spinning is not completed. It is interesting to note that in spite of different ways of determination, the volume fractions of the Cu_2_MnAl phase in the sample aged for 6 h and that in the sample aged for 12 h are close to each other and correspond to the data from [3].

**Fig. 5:**
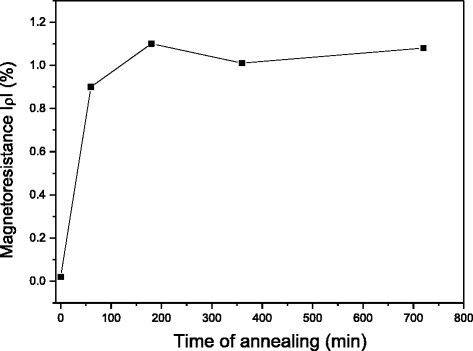
Magnetoresistance at room temperature as a function of annealing time for Cu-7 % Mn-26 % Al alloy

According to the theoretical model [7], the magnetoresistance of the granular system is proportional to the square of the magnetization: *ρ* ~ −*α*(*M*/*M*_S_)^2^ where *α* is a constant . Changing time of aging, one can vary particle size and the volume fraction of ferromagnetic phase. These parameters affect scattering of electrons on the interfaces between particle and matrix and inside particle as well as affect magnetization. In Fig. 5, absolute values of the magnetoresistance in the field of 6 kOe are plotted against time of aging. It follows from the plot that increasing aging time up to 3 h leads to a sharp increase of magnetoresistance while longer thermal treatment is not effective. The maximal magnitude of magnetoresistance for Cu-7 % Mn-26 % Al alloy achieves 1.1 %. The results obtained suggest that the fast growth of the magnetoresistance with aging time terminates as formation of the dispersed structure comes to an end and the volume fraction of ferromagnetic nanoparticles no longer increases.

## Conclusions

Results presented in this work demonstrate that magnetic and transport properties of the Cu-Mn-Al ribbons closely correlate with parameters of precipitated nanoparticles formed during solid solution decomposition. The ribbons of the Cu-5 % Mn-23.4 % Al composition do not show presence of coherent nanoparticles according to X-ray diffraction analysis even after aging for 2 h. It can be explained by a small size of them. This results in low magnetoresistance that does not exceed 0.1 %.

The ribbons of the Cu-7 % Mn-26 % Al composition demonstrate superparamagnetic behavior at room temperature irrespective of aging time. The estimations using magnetization curves fitted to Langevin function allowed for determination of the average particle size, the interparticle spacing, and the concentration of ferromagnetic nanoparticles. The volume fractions of Cu_2_MnAl nanoparticles in the Cu-7 % Mn-26 % Al ribbons demonstrated growth from 0.09 to 0.2 after the melt-spun ribbons were aged for 6 h. Aging also affected the magnetoresistance, increasing it with time during the first 3 h of the process. Magnetization measurements also revealed that freezing of magnetic moments at low temperatures characteristic of both the as-spun and the aged ribbons is most likely caused by the dipole-dipole interaction.

## References

[1] Shi J, Gider S, Babcock K, Awschalom DD (1996). Magnetic clusters in molecular beams, metals, and semiconductors. Science.

[2] Mydosh JA (1993). Spin glasses: an experimental introduction.

[3] Bouchard M, Thomas G (1975). Phase transition and modulated structures in ordered (Cu-Mn)_3_Al alloys. Acta Metall..

[4] Yiping L, Murthy A, Hadjipanayis GC, Wan H (1996). Giant magnetoresistance in Cu-Mn-Al. Phys. Rev. B.

[5] Kokorin VV (1987). Martensitnye Prevrashchenia v Neodnorodnykh Tverdykh Rasvorakh (Martensitic transformations in inhomogeneous solid solutions).

[6] Robinson J, McCormick P, Street R (1995). Structure and properties of Cu_2_MnAl synthesized by mechanical alloying. J. Phys. Condens. Matt..

[7] Zhang S (1992). Theory of giant magnetoresistance in magnetic granular films. Appl. Phys. Lett..

